# 中国非小细胞肺癌免疫检查点抑制剂治疗专家共识（2020年版）

**DOI:** 10.3779/j.issn.1009-3419.2021.101.13

**Published:** 2021-04-20

**Authors:** 彩存 周, 洁 王, 宝成 王, 颖 程, 哲海 王, 宝惠 韩, 铀 卢, 钢 伍, 力 张, 勇 宋, 波 朱, 毅 胡, 子平 王, 启斌 宋, 胜祥 任, 雅億 何, 晓桦 胡, 艰 张, 煜 姚, 洪云 赵, 志杰 王, 倩 褚, 建春 段, 菁菁 柳, 叔逵 秦

**Affiliations:** 1 200433 上海，同济大学附属上海市肺科医院 Shanghai Pulmonary Hospital, Tongji University School of Medicine, Shanghai 200433, China; 2 100021 北京，国家癌症中心/国家肿瘤临床医学研究中心/中国医学科学院 & 北京协和医学院肿瘤医院 National Cancer Center/National Clinical Research Center for Cancer/Cancer Hospital, Chinese Academy of Medical Sciences and Peking Union Medical College, Beijing 100021, China; 3 250031 济南，中国人民解放军联勤保障部队第960医院 No. 960 Hospital of PLA, Jinan 250031, China; 4 130012 长春，吉林省肿瘤医院 Jilin Cancer Hospital, Changchun 130012, China; 5 250117 济南，山东省肿瘤医院 Shandong Cancer Hospital and Institute, Jinan 250117, China; 6 200030 上海，上海市胸科医院 Shanghai Chest Hospital, Shanghai 200030, China; 7 610041 成都，四川大学华西医院 West China Hospital, Sichuan University, Chengdu 610041, China; 8 430022 武汉，华中科技大学同济医学院附属协和医院 Union Hospital, Tongji Medical College, Huazhong University of Science and Technology, Wuhan 430022, China; 9 100010 北京，北京协和医院 Peking Union Medical College Hospital, Beijing 100010, China; 10 210002 南京，东部战区总医院 General Hospital of Eastern Theater Command, Nanjing 210002, China; 11 400037 重庆，重庆新桥医院 Xinqiao Hospital, The Army Medical University, Chongqing 400037, China; 12 100853 北京，中国人民解放军总医院 Chinese PLA General Hospital, Beijing 100853, China; 13 100142 北京，北京大学肿瘤医院 Beijing Cancer Hospital, Beijing 100142, China; 14 430060 武汉，武汉大学人民医院 Renmin Hospital of Wuhan University, Wuhan 430060, China; 15 530021 南宁，广西医科大学第一附属医院 The First Affiliated Hospital of Guangxi Medical University, Nanning 530021, China; 16 710032 西安，西京医院 Xijing Hospital, Xi'an 710032, China; 17 710061 西安，西安交通大学第一附属医院 The First Affiliated Hospital of Xi'an Jiaotong University, Xi'an 710061, China; 18 510060 广州，中山大学肿瘤防治中心 Sun Yat-sen University Cancer Center, Guangzhou 510060, China; 19 430030 武汉，华中科技大学同济医学院附属同济医院 Tongji Hospital of Tongji Medical College, Huazhong University of Science and Technology, Wuhan 430030, China; 20 210002 南京，解放军东部战区总医院肿瘤中心 Cancer Center, Eastern Theater General Hospital of the Chinese PLA, Nanjing 210002, China

**Keywords:** 肺肿瘤, 免疫治疗, 程序性死亡分子-1/程序性死亡分子配体-1, 专家共识, Lung neoplasms, Immunotherapy, Programmed cell death protein 1/Programmed death-ligand 1, Expert consensus

## Abstract

非小细胞肺癌（non-small cell lung cancer, NSCLC）是肺癌最常见的病理类型。晚期NSCLC的系统性抗肿瘤治疗经历了化疗、靶向治疗及免疫治疗的变革，患者总体生存时间不断延长。免疫检查点抑制剂（immune checkpoint inhibitors, ICIs），尤其是程序性死亡分子-1（programmed cell death protein 1, PD-1）/程序性死亡分子配体-1（programmed death-ligand 1, PD-L1）抗体已成为表皮生长因子受体（epidermal growth factor receptor, EGFR）/间变性淋巴瘤激酶（anaplastic lymphoma kinase, ALK）阴性晚期NSCLC一线及二线的标准治疗和局部晚期NSCLC同步放化疗后标准治疗，并在辅助/新辅助治疗中显示出可喜的结果，改变了NSCLC整体治疗格局。随着越来越多的ICIs在国内获批肺癌适应证，中国临床肿瘤学会（Chinese Society of Clinical Oncology, CSCO）NSCLC专家委员会牵头，组织该领域的专家，结合2019年版专家共识，参考最新国内外文献、临床研究数据及系统评价，在专家共同讨论的基础上，达成统一意见并制定、更新本共识，为国内同行更好地应用ICIs治疗NSCLC提供参考意见。

## Abstract (en)

Non-small cell lung cancer (NSCLC) is the most common pathological type of lung cancer. The systemic antitumor therapy of advanced NSCLC has undergone renovations of chemotherapy, targeted therapy and immunotherapy, which results in greatly improved survival for patients with advanced NSCLC. Immune checkpoint inhibitors (ICIs), especially targeting programmed cell death protein 1 (PD-1)/programmed death-ligand 1 (PD-L1), has changed the treatment paradigm of NSCLC. ICIs have become the standard treatment for advanced NSCLC without epidermal growth factor receptor(EGFR) mutation or anaplastic lymphomakinase(ALK) translocation in the first- or second-line setting, and for locally advanced NSCLC following concurrent radiotherapy and chemotherapy. ICIs are also promising in adjuvant/neoadjuvant therapy. More and more ICIs have been approved domestically for the treatment of NSCLC. Led by the NSCLC expert committee of Chinese Society of Clinical Oncology (CSCO), this consensus was developed and updated based on thoroughly reviewing domestic and foreign literatures, clinical trial data, systematic reviews, experts' discussion and the consensus(2019 version). This consensus will aid domestic clinicians in the treatment of NSCLC with ICIs.

## 1 前言

肺癌是全球发病率第二、死亡率第一的恶性肿瘤^[1, 2]^。据2020年全球肿瘤流行病统计数据GLOBOCAN分析报告显示，全球肺癌新发例数达220.7万，仅次于乳腺癌；死亡例数达179.6万，居各癌种首位。在我国，肺癌不仅是发病率第一，也是死亡率第一的恶性肿瘤^[2, 3]^。2020年我国肺癌预估新发病例达81.6万，占所有新发癌症的17.9%，死亡例数达71.5万，占癌症总死亡人数的23.8%^[4]^。按年龄标化率（age standardized rate, ASR）预计，2020年我国肺癌男女发病率分别为ASR 47.8/10万和22.8/10万，死亡率分别为ASR 41.8/10万和19.7/10万^[4]^。肺癌可分为非小细胞肺癌（non-small cell lung cancer, NSCLC）和小细胞肺癌两大病理组织学类型，其中NSCLC最为常见，占所有肺癌的85%^[5]^。对于驱动基因阴性晚期NSCLC，传统含铂双药化疗的中位无进展生存期（median progression free survival, mPFS）仅4个月-6个月，中位总生存期（median overall survival, mOS）仅10个月-12个月^[6-9]^，而免疫治疗能为驱动基因阴性的晚期NSCLC带来生存获益。研究者^[10]^预测，免疫治疗的出现将会进一步改善肺癌患者的生存结局，尤其是驱动基因突变阴性晚期NSCLC。

免疫治疗自20世纪90年代问世以来，已在癌症治疗领域取得了突破性进展，2013年被《Science》杂志评为年度十大科学突破之首^[11]^。美国食品药品监督管理局（Food and Drug Administration, FDA）与中国国家药品监督管理局（National Medical Products Administration, NMPA）相继于2015年和2018年批准首个免疫检查点抑制剂（immune checkpoint inhibitors, ICIs）用于肺癌治疗。随着越来越多的ICIs在我国获批肺癌适应证，免疫治疗在肺癌治疗领域的应用逐步普及。在临床实践中，如何选择优势人群、确定治疗方案、评估疗效、处理不良反应（adverse event, AE）以及考量药物使用禁忌证等方面的临床标准仍需不断完善。因此，中国临床肿瘤学会（Chinese Society of Clinical Oncology, CSCO）NSCLC专家委员会牵头，组织肺癌领域的专家，在参考最新的国内外文献、临床研究数据及系统评价、在专家共同讨论的基础上，形成共识并更新《中国非小细胞肺癌免疫检查点抑制剂治疗专家共识（2020年版）》，供国内同行参考，以期进一步规范NSCLC免疫治疗的临床实践。

## 2 肿瘤的免疫逃逸机制

### 2.1 机体正常的免疫监视

在正常生理状态下，免疫系统具有“监视功能”，可精确识别“非己”成分；免疫系统可清除入侵的微生物，排斥异体移植物，还能察觉并消灭肿瘤细胞。随着免疫学研究的深入，已经证实，T淋巴细胞介导的细胞免疫是抗肿瘤免疫的主要机制。肿瘤细胞产生的新抗原被抗原递呈细胞（antigen presenting cells, APCs）摄取并加工处理，并以抗原肽-主要组织相容性复合体（major histocompatibility complex, MHC）Ⅰ类/Ⅱ类分子复合物的形式由APCs提呈给T细胞，使得T细胞活化、增殖、分化成肿瘤抗原特异性细胞毒性T细胞（cytotoxic T cell, CTL）。随后CTL迁移并浸润肿瘤床，并通过T细胞受体（T cell receptor, TCR）和抗原肽-MHC Ⅰ类分子复合物之间的相互作用，特异性识别并杀死肿瘤细胞。肿瘤细胞凋亡后又会释放更多的肿瘤相关抗原，进一步激活更多的T细胞以进一步杀伤肿瘤^[12]^。

### 2.2 肿瘤的免疫逃逸机制和相应的治疗策略

正常情况下，机体免疫监视功能可识别并清除癌变的细胞，但肿瘤细胞有多种方法逃避免疫系统监视，最终导致肿瘤的发生和发展。肿瘤免疫逃逸机制大致可分为以下三个方面^[13]^：①免疫检查点：肿瘤细胞除了表达可被免疫系统识别的某些特殊抗原，还可表达多种免疫抑制性配体，与T细胞表达的抑制性受体[程序性死亡分子-1（programmed cell death protein 1, PD-1）、LAG-3、TIM-3、TIGIT、VISTA、CD244等]结合，这些共抑制分子会抑制T细胞功能。可通过阻断共抑制信号解除对T细胞的活化和增殖的抑制，从而杀死肿瘤细胞^[13]^。②抗原性丧失：肿瘤细胞通过丧失特异性抗原的表达来避开免疫系统的识别，从而躲避免疫系统的监视。可通过细胞工程技术改造免疫细胞，使之可以识别肿瘤细胞表面的其他特定“非己”抗原而杀死肿瘤细胞^[14, 15]^。③免疫抑制微环境：在实体肿瘤组织内，存在多种负性调节的细胞和细胞因子，共同构成了肿瘤组织周围的免疫抑制性微环境。可通过调节免疫细胞及其分泌因子，促进血管生成等多重参与肿瘤微环境的因素实现免疫治疗^[16]^。

## 3 免疫检查点抑制剂

T细胞活化时，相应共抑制信号通路的免疫检查点，包括PD-1/程序性死亡分子配体-1（programmed death-ligand 1, PD-L1）和细胞毒性T淋巴细胞相关抗原4（cytotoxic T lymphocyte antigen 4, CTLA-4）的表达会增加，而ICIs通过阻断上述检查点恢复或增强机体的抗肿瘤免疫^[17]^。

### 3.1 PD-（L）1抑制剂

PD-1是表达在T细胞表面的一种重要的免疫抑制跨膜蛋白，其主要配体为PD-L1。肿瘤细胞能够表达PD-L1，与PD-1结合，导致PD-1胞质域的酪氨酸磷酸化和酪氨酸磷酸酶SHP-2的募集，使得T细胞受体（T cell receptor, TCR）信号分子去磷酸化，减弱了TCR下游的信号激活，降低了T细胞活化和细胞因子生成^[18]^。PD-（L）1抑制剂正是通过阻断PD-1/L1的结合，恢复机体对肿瘤细胞的免疫杀伤功能。PD-（L）1抑制剂见表 1。

**表 1:** NSCLC治疗领域已上市或公布Ⅲ期研究结果的PD-（L）1抑制剂产品列表 List of PD-(L) 1 agent approved on the market or released results of phase Ⅲ clinical trials

类型通用名	PD-1						PD-L1		
	帕博利珠单抗	纳武利尤单抗	信迪利单抗	卡瑞利珠单抗	替雷利珠单抗		度伐利尤单抗	阿替利珠单抗	舒格利单抗
通用名英文	Pembrolizumab	Nivolumab	Sintilimab	Camrelizumab	Tislelizumab		Durvalumab	Atezolizumab	Sugemalimab
商品名	可瑞达	欧狄沃	达伯舒	艾瑞卡	百泽安		英飞凡	泰圣奇	/
商品名英文	Keytruda	Opdivo	Tyvyt	/	/		Imfinzi	Tecentriq	/
生产商	默沙东	百时美施贵宝	信达生物	恒瑞医药	百济神州		阿斯利康	罗氏	基石
抗体类型	人源化IgG4	全人源IgG4	全人源IgG4	人源化IgG4	人源化IgG4		全人源IgG1	人源化IgG1	全人源IgG4
NMPA获批	一线	二线	一线非鳞癌	一线	一线鳞癌		Ⅲ期不可切	尚未获批	尚未获批
	鳞癌及非鳞癌	鳞癌及非鳞癌		非鳞癌			NSCLC		
FDA获批	一线及二线	一线至二线	尚未获批	尚未获批	尚未获批		Ⅲ期不可切	一线及二线	尚未获批
	鳞癌及非鳞癌	鳞癌及非鳞癌					NSCLC	鳞癌/非鳞癌	
PD-1：程序性死亡分子-1；PD-L1：程序性死亡分子配体-1；NSCLC：非小细胞肺癌；NMPA：国家药品监督管理局；FDA：美国食品药品监督管理局									

### 3.2 CTLA-4抑制剂

CTLA-4是由CTLA-4基因编码的一种跨膜蛋白，表达于活化的CD4^+^和CD8^+^ T细胞，与配体CD80（B7-1）和CD86（B7-2）结合。CTLA-4可通过竞争性抑制CD28与B7配体结合或将磷酸酶募集到CTLA-4的胞质域，降低TCR和CD28信号传导，抑制T细胞活化。其次，CTLA-4可通过细胞因子下调APC上CD80/CD86的表达，或通过胞吞作用，使CD80/CD86移出APC，从而减少CD28与B7配体的结合。再次，CTLA-4还能通过结合树突状细胞表达的CD80/CD86并诱导色氨酸降解酶吲哚胺2, 3-双加氧酶（indoleamine 2, 3-dioxygenase, IDO）的表达，从而抑制T细胞应答^[18]^。目前国内尚无CTLA-4抗体获批上市，国外已批准CTLA-4抗体有伊匹单抗（Ipilimumab，商品名“Yervoy”）用于治疗晚期NSCLC等。

## 4 NSCLC的免疫治疗

### 4.1 驱动基因突变阴性NSCLC

#### 4.1.1 晚期NSCLC一线免疫治疗

见表 2。

**表 2:** 晚期NSCLC一线免疫治疗★ First-line immunotherapy for advanced NSCLC★

分层	非鳞癌				鳞癌		
	一级推荐	二级推荐	三级推荐		一级推荐	二级推荐	三级推荐
任意PD-L1	帕博利珠单抗联合铂类+培美曲塞	阿替利珠单抗联合贝伐珠单抗联合卡铂+紫杉醇	纳武利尤单抗联合伊匹单抗联合铂类+培美曲塞		帕博利珠单抗联合铂类+紫杉类	信迪利单抗联合铂类+吉西他滨	纳武利尤单抗联合伊匹单抗联合卡铂+紫杉醇
	卡瑞利珠单抗联合卡铂+培美曲塞	阿替利珠单抗联合卡铂+白蛋白紫杉醇			替雷利珠单抗联合铂类+紫杉类		
	信迪利单抗联合铂类+培美曲塞	替雷利珠单抗联合铂类+培美曲塞					
PD-L1≥50%	帕博利珠单抗单药^※^	阿替利珠单抗单药			帕博利珠单抗单药^※^	阿替利珠单抗单药	
1%≤PD-L1≤49%	帕博利珠单抗单药^*^		纳武利尤单抗联合伊匹单抗^#^		帕博利珠单抗单药^*^		纳武利尤单抗联合伊匹单抗^#^
★本专家共识推荐说明： 1.一级推荐为目前国内已商业上市且有NMPA获批适应证的药物； 2.二级推荐是国内已商业上市且有充分证据的药物，但目前尚未取得NMPA获批相关适应证； 3.三级推荐是国内尚未商业上市，但在国外获批或者有充分证据的数据。 ^※^PD-L1≥50%的人群，帕博利珠单抗单药显著优于含铂双药化疗（KEYNOTE-024/042），帕博利珠单抗单药尤其适合肿瘤负荷小、疾病进展缓慢的PD-L1高表达患者。 ^*^PD-L1 1%-49%的人群，使用帕博利珠单抗单药和化疗疗效相当，mOS分别为13.4个月*vs* 12.1个月（HR=0.90; 95%CI: 0.77-1.06）（KEYNOTE-042），提示对于化疗禁忌证患者，帕博利珠单抗单药可作为一种选择。 ^#^KEYNOTE-598研究结果为阴性，谨慎推荐和使用PD-（L）1+CTLA-4双免治疗模式。mOS：中位总生存期；CTLA-4：细胞毒性T淋巴细胞相关抗原4							

主要证据来源：①免疫联合化疗：不论PD-L1表达：非鳞癌：帕博利珠单抗联合培美曲塞/铂类（KEYNOTE-021G, KEYNOTE-189），FDA与NMPA批准；阿替利珠单抗联合白蛋白紫杉醇/卡铂（IMpower130），FDA批准；卡瑞利珠单抗联合培美曲塞/卡铂（CameL），NMPA批准；信迪利单抗联合培美曲塞/铂类（ORIENT-11），NMPA批准；替雷利珠单抗联合培美曲塞/铂类（RATIONALE 304）；舒格利单抗联合培美曲塞/卡铂（GEMSTONE-302）；鳞癌：帕博利珠单抗联合紫杉醇或白蛋白紫杉醇/卡铂（KEYNOTE-407），FDA与NMPA批准；替雷利珠单抗联合紫杉醇或白蛋白紫杉醇/卡铂（RATIONALE 307），NMPA批准；信迪利单抗联合吉西他滨/铂类（ORIENT-12）；舒格利单抗联合紫杉醇/卡铂（GEMSTONE-302）；②单药：帕博利珠单抗（KEYNOTE-024，KEYNOTE-042），PD-L1≥1%，FDA和NMPA批准；阿替利珠单抗（IMpower110），TC3/IC3人群，FDA批准；③免疫联合化疗和抗血管：阿替利珠单抗+贝伐珠单抗+紫杉醇+卡铂（IMpower150），FDA批准；④双免疫联合治疗：纳武利尤单抗联合伊匹单抗（CheckMate 227），PD-L1≥1%，FDA批准；⑤双免疫联合化疗：纳武利尤单抗+伊匹单抗+2个周期含铂化疗（CheckMate 9LA），FDA批准（表 3）。

**表 3:** ICIs一线治疗晚期NSCLC Ⅲ期临床研究结果汇总 Summary of results from phase Ⅲ clinical trials of ICIs as first-line therapy for advanced NSCLC

治疗模式	病理学类型	研究	样本量	PD-L1表达	治疗组	mOS（mon）	生存率^※^	mPFS（mon）	ORR
免疫联合化疗	非鳞癌	KEYNOTE-189^[19, 20]^	616	/	帕博利珠单抗+化疗*vs*化疗	*22.0 *vs* 10.6 (HR=0.60)	31.3% *vs* 17.4% (3年)	*9.0 *vs* 4.9 (HR=0.50)	48.3% *vs* 19.9%
		IMpower130^[21]^	724	/	阿替利珠单抗+化疗*vs*化疗	*18.6 *vs* 13.9 (HR=0.79)	39.6% *vs* 30% (2年)	*7.0 *vs* 5.5 (HR=0.64)	49.2% *vs* 31.9%
		CameL^[22, 23]^	412	/	卡瑞利珠单抗+化疗*vs*化疗	27.9 *vs* 20.5 (HR=0.73)	/	*11.3 *vs* 8.3 (HR=0.60)	60.5% *vs* 38.6%
		ORIENT-11^[24]^	397	/	信迪利单抗+化疗*vs*化疗	NR *vs* NR (HR=0.609)	/	*8.9 *vs* 5.0 (HR=0.482)	51.9% *vs* 29.8%
		RATIONALE 304^[25]^	334	/	替雷利珠单抗+化疗*vs*化疗	/	/	*9.7 *vs* 7.6 (HR=0.645)	57.4% *vs* 36.9%
		GEMSTONE-302^[26]^	287	/	舒格利单抗+化疗*vs*化疗	NR *vs* 14.75 (HR=0.66)	/	*8.57 *vs* 5.16 (HR=0.66)	56.2% *vs* 34.7%
	鳞癌	KEYNOTE-407^[27-29]^	559	/	帕博利珠单抗+化疗*vs*化疗	*17.1 *vs* 11.6 (HR=0.71)	37.5% *vs* 30.6% (2年)	*8.0 *vs* 5.1 (HR=0.57)	62.6% *vs* 38.4%
		KEYNOTE-407 China Study^[30]^	125	/	帕博利珠单抗+化疗*vs*化疗	*17.3 *vs* 12.6 (HR=0.44)	/	*8.3 *vs* 4.2 (HR=0.32)	78.5% *vs* 41.7%
		RATIONALE 307^[31]^	360	/	替雷利珠单抗+化疗（紫杉醇或白蛋白紫杉醇/卡铂）*vs*化疗	NR, NR, NR	/	*7.6, 7.6 *vs* 5.5 (HR=0.524; 0.478)	72.5%, 74.8% *vs* 49.6%
		ORIENT-12^[32]^	357	/	信迪利单抗+化疗*vs*化疗	NR *vs* NR (HR=0.567)	/	*5.5 *vs* 4.9 (HR=0.536)	44.7% *vs* 35.4%
		GEMSTONE-302^[26]^	192	/	舒格利单抗+化疗*vs*化疗	NR *vs* 14.75 (HR=0.66)	/	*7.16 *vs* 4.70 (HR=0.33)	69.0% *vs* 46.0%
单药	非鳞癌 & 鳞癌	KEYNOTE-024^[33, 34]^	305	≥50%	帕博利珠单抗*vs*化疗	26.3 *vs* 13.4 (HR=0.62)	31.9% *vs* 16.3% (5年)	*10.3 *vs* 6.0 (HR=0.50)	46.1% *vs* 31.1%
		KEYNOTE-042^[35]^	1, 274	≥1%	帕博利珠单抗*vs*化疗	*16.4 *vs* 12.1 (HR=0.80)	25% *vs* 17% (3年)	5.5 *vs* 6.8 (HR=1.05)	27.3% *vs* 26.7%
			599	≥50%		*20.0 *vs* 12.2 (HR=0.68)	31% *vs* 18% (3年)	6.5 *vs* 6.5 (HR=0.85)	39.1% *vs* 32.3%
		KEYNOTE-042 China Study^[36]^	262	≥1%	帕博利珠单抗*vs*化疗	*20.2 *vs* 13.5 (HR=0.67)	43.8% *vs* 28.2% (2年)	6.3 *vs* 6.7 (HR=1.0)	31.3% *vs* 24.6%
			146	≥50%		*24.5 *vs* 13.8 (HR=0.63)	50.0% *vs* 29.7% (2年)	8.3 *vs* 6.5 (HR=0.84)	40.3% *vs* 24.3%
		IMpower110^[37, 38]^	554	IC3/TC3	阿替利珠单抗*vs*化疗	*20.2 *vs* 14.7 (HR=0.76)	/	8.2 *vs* 5.0 (HR=0.59)	40.2% *vs* 28.6%
		CheckMate 026^[39]^	423	≥5%	纳武利尤单抗*vs*化疗	14.4 *vs* 13.2 (HR=1.02)	/	*4.2 *vs* 5.9 (HR=1.15)	26% *vs* 33%
联合化疗和抗血管	非鳞癌	IMpower150^[40, 41]^	1, 202	/	阿替利珠单抗+化疗+贝伐珠单抗*vs*化疗+贝伐珠单抗	*19.5 *vs* 14.7 (HR=0.80)	43.4% *vs* 33.7% (2年)	*8.3 *vs* 6.8 (HR=0.62)	63.5% *vs* 48.0%
双免联合	非鳞癌 & 鳞癌	CheckMate 227^[42]^	1, 189	≥1%	纳武利尤单抗+伊匹单抗*vs*化疗	*17.1 *vs* 14.9 (HR=0.79)	33% *vs* 22% (3年)	*5.1 *vs* 5.6 (HR=0.81)	36.4% *vs* 30.2%
		KEYNOTE-598^[43]^	568	≥50%	帕博利珠单抗+伊匹单抗*vs*帕博利珠单抗	*21.4 *vs* 21.9 (HR=1.08)	/	*8.2 *vs* 8.4 (HR=1.06)	45.4% *vs* 45.4%
双免联合化疗	非鳞癌 & 鳞癌	CheckMate 9LA^[44]^	719	/	纳武利尤单抗+伊匹单抗+化疗*vs*化疗	*15.6 *vs* 10.9 (HR=0.66)	/	6.7 *vs* 5.0 (HR=0.68)	38% *vs* 25%
mPFS：中位无进展生存期；ORR：客观缓解率。^*^主要研究终点；^※^仅显示≥2年的生存率。临床研究释义详见附录。									

#### 4.1.2 晚期NSCLC二线免疫治疗

见表 4。

**表 4:** 晚期NSCLC二线免疫治疗 Second-line immunotherapy for advanced NSCLC

分层	一级推荐	二级推荐
既往无PD-（L）1抑制剂治疗	纳武利尤单抗单药	帕博利珠单抗单药（PD-L1≥1%）； 阿替利珠单抗单药
既往有PD-（L）1抑制剂治疗	既往PD-（L）1抑制剂治疗： 含铂两药联合化疗方案（根据组织学类型选择合适的化疗方案或联合贝伐珠单抗）	
	既往PD-（L）1抑制剂联合化疗治疗： 多西他赛或其他单药化疗（一线未曾接受过的药物）	

主要证据来源：①纳武利尤单抗单药（CheckMate 017, CheckMate 057, CheckMate 078），不论PD-L1表达，FDA与NMPA批准；②帕博利珠单抗单药（KEYNOTE-010），PD-L1≥1%，FDA批准；③阿替利珠单抗单药（OAK），不论PD-L1表达，FDA批准（表 5）。

**表 5:** ICIs二线治疗晚期NSCLC临床研究结果汇总 Summary of results from phase Ⅲ clinical trials of ICIs as second-line therapy for advanced NSCLC

治疗模式	病理学类型	研究	样本量	PD-L1表达	治疗组	mOS（mon）	生存率	mPFS（mon）	ORR
单药	非鳞癌 & 鳞癌	KEYNOTE- 010^[45, 46]^	1, 033	≥50%	帕博利珠单抗*vs* Doc	*16.9 *vs* 8.2 (HR=0.55)	25.0% *vs* 8.2% (5年)	*5.3 *vs* 4.2 (HR=0.57)	33.1% *vs* 9.2%
			442	≥1%		*11.8 *vs* 8.4 (HR=0.70)	15.6% *vs* 6.5% (5年)	*4.0 *vs* 4.1 (HR=0.84)	21.2% *vs* 9.6%
		OAK^[47, 48]^	1, 225	/	阿替利珠单抗*vs* Doc	*13.3 *vs* 9.8 (HR=0.78)	16% *vs* 9% (4年)	2.8 *vs* 4.0 (HR=0.95)	14% *vs* 13%
		CheckMate 078^[49, 50]^	504	/	纳武利尤单抗*vs* Doc	*11.9 *vs* 9.5 (HR=0.75)	19% *vs* 12% (3年)	2.8 *vs* 2.8 (HR=0.78)	18% *vs* 4%
	鳞癌	CheckMate 017^[51, 52]^	272	/	纳武利尤单抗*vs* Doc	*9.2 *vs* 6.0 (HR=0.62)	12.3% *vs* 3.6% (5年)	3.5 *vs* 2.8 (HR=0.62)	20% *vs* 9%
	非鳞癌	CheckMate 057^[52, 53]^	582	/	纳武利尤单抗*vs* Doc	*12.2 *vs* 9.5 (HR=0.70)	14.0% *vs* 2.1% (5年)	2.3 *vs* 4.2 (HR=0.92)	19% *vs* 12%
Doc：多西他赛；*主要研究终点。临床研究释义详见附录。									

#### 4.1.3 Ⅲ期不可切除的NSCLC免疫治疗

见表 6。

**表 6:** Ⅲ期不可切除的NSCLC免疫治疗 Immunotherapy for stage Ⅲ non-resectable NSCLC

分层	一级推荐
适合放化疗	根治性同步放化疗→度伐利尤单抗巩固治疗

主要证据来源：度伐利尤单抗用于不可切除Ⅲ期NSCLC同步放化疗后的巩固治疗（PACIFIC），FDA和NMPA批准。

PACIFIC研究在接受含铂同步放化疗后未发生疾病进展的Ⅲ期不可切除的NSCLC患者中评估了度伐利尤单抗巩固治疗对比安慰剂的疗效。结果显示，度伐利尤单抗对比安慰剂治疗显著延长OS（47.5个月*vs* 29.1个月，HR=0.71）和生存率（4年：49.6% *vs* 36.3%）^[54, 55]^。目前FDA和NMPA批准度伐利尤单抗用于不可切除Ⅲ期NSCLC同步放化疗后的巩固治疗。

### 4.2 驱动基因突变阳性NSCLC

对于驱动基因阳性的NSCLC进行免疫治疗目前尚缺乏充分证据。IMpower150是第一个在亚组中显示ICIs对表皮生长因子受体（epidermal growth factor receptor, *EGFR*）阳性患者有临床获益的研究。针对*EGFR*阳性亚组的进一步分析显示，阿替利珠单抗+贝伐珠单抗+化疗组较贝伐珠单抗联合化疗组具有PFS获益（10.2个月*vs*7.1个月，HR=0.56），且OS获益在*EGFR*敏感突变NSCLC患者中更明显（29.4个月*vs*18.1个月，HR=0.60）^[56]^。CT18 Ⅱ期临床研究中探索了特瑞普利单抗联合化疗用于EGFR-TKI治疗失败的*EGFR*突变阳性T790M阴性晚期NSCLC患者的疗效和安全性，客观缓解率（objective response rate, ORR）达50.0%，mPFS达7.0个月^[57]^。针对*EGFR*阳性晚期NSCLC，国内外免疫联合抗血管生成或化疗的多项研究（KEYNOTE-789、Checkmate-722、ORIENT-31和TREASURE）正在进行中，有望探索出新的治疗方案。

### 4.3 早期NSCLC的新辅助/辅助免疫治疗

免疫治疗也在早期NSCLC患者中进行着积极的探索。目前多项Ⅰ期/Ⅱ期研究的结果显示了良好的免疫应答和临床获益，尽管这些研究的入组人群、治疗方案、治疗周期等存在差异^[58]^。CheckMate 159、LCMC3、PRINCEPS、TOP1501、IoNESCO及ChicTR-OIC-17013726等免疫单药新辅助研究入组Ⅰ期-Ⅲb期NSCLC患者，治疗1个-3个周期，病理学显著缓解率（major pathologic response, MPR）为14%-45%^[59-62]^。免疫联合化疗（NADIM、NCT02716038、SAKK 16/14）新辅助研究入组Ib期-Ⅲa期的患者，治疗2个-4个周期，其中，NADIM的MPR达到85.36%，病理学完全缓解（pathologic complete response, pCR）达到71.4%，其余两项研究的MPR在60%左右^[63-65]^。双免（NEOSTAR）新辅助纳入Ⅰ期-Ⅲa期的患者，治疗3个周期，MPR率为24%^[66]^。免疫新辅助治疗后手术延迟率低，总体平均手术切除率为88.70%，未增加手术难度及围术期风险，手术并发症的平均发生率为20.6%，多数预后良好^[58]^。KEYNOTE-671、CheckMate 816、IMpower030、Checkmate 77T、AEGEAN等多项Ⅲ期研究也在进行中。

## 5 生物标志物

### 5.1 PD-L1表达

目前认为肿瘤组织PD-L1表达是PD-（L）1抑制剂治疗前选择优势人群标准的生物标志物。KEYNOTE-024研究结果显示帕博利珠单抗在PD-L1肿瘤比例评分（tumor proportion score, TPS）≥50%的驱动基因阴性的晚期NSCLC人群中，一线治疗效果优于化疗^[33]^。KEYNOTE-042研究显示帕博利珠单抗能显著改善PD-L1 TPS≥1% NSCLC患者的mOS^[67]^。IMpower110和Empower-Lung 1研究也显示了类似的结果，这四项临床研究均证实了PD-L1表达水平与免疫治疗疗效的相关性。CheckMate 057研究对比了纳武利尤单抗单药与多西他赛二线治疗NSCLC的疗效，无论PD-L1的表达水平，免疫治疗相较于化疗均能获益，但在PD-L1低表达或不可检测的患者中，未观察到相似的OS获益^[53]^。因此PD-L1是晚期NSCLC的免疫治疗疗效预测的生物标志物之一，NCCN及CSCO指南推荐PD-L1检测结果可以作为伴随诊断指导晚期NSCLC患者一线接受免疫治疗。

### 5.2 肿瘤基因突变负荷（tumor mutational burden, TMB）/血液TMB（blood TMB, bTMB）

CheckMate 026^[68]^探索性分析提示高TMB患者可从免疫治疗中获益。KEYNOTE系列研究的探索性分析显示，无论TMB高低，帕博利珠单抗联合化疗一线治疗使NSCLC患者生存获益^[27]^。KEYNOTE-158研究评估泛实体瘤患者中TMB高（TMB-high, TMB-H）与帕博利珠单抗疗效的关系，TMB-H患者ORR达29%，非TMB-H仅6%^[69]^。2020年FDA批准了帕博利珠单抗单药治疗TMB-H且既往治疗后疾病进展的无法切除或转移性实体瘤患者，同时也批准FoundationOne CDx作为治疗的伴随诊断。如组织标本不足，利用ctDNA检测bTMB潜在可行^[70, 71]^。

### 5.3 错配修复缺陷（mismatch repair deficient, dMMR）/微卫星不稳定-高（microsatellite instability-high, MSI-H）及其他基因变异

帕博利珠单抗被FDA批准用于治疗MSI-H/dMMR的所有实体瘤患者^[72]^。CheckMate 142研究^[73]^显示纳武利尤单抗单药联或不联合伊匹单抗治疗MSI-H的转移性结直肠癌有一定疗效。dMMR/MSI-H在肺癌中的发生率很低^[74]^，故其对肺癌免疫治疗疗效的预测价值还需进一步验证。其他一些生物标志物，如*KRAS*、*STK11*、*KEAP1*等基因以及DNA应答通路（DNA damage response, DDR）基因变异^[75]^对免疫治疗疗效的预测价值目前仍在探索之中，不作为常规推荐。

### 5.4 晚期NSCLC的治疗路径图

见图 1。

**图 1:**
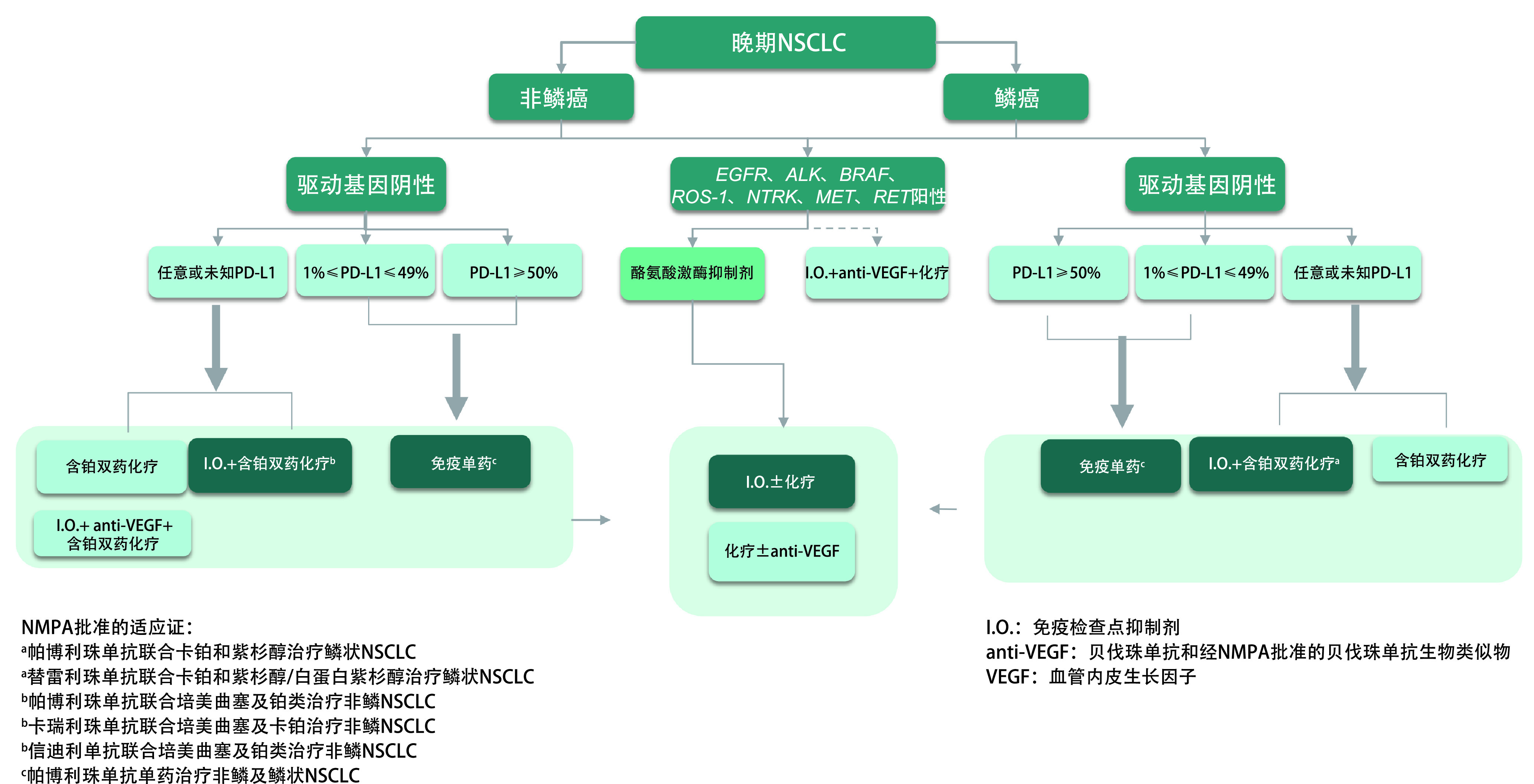
晚期NSCLC的治疗路径图 Treatment pathway for advanced NSCLC

## 6 免疫治疗的评估

目前，对于肿瘤治疗疗效的评价，通常采用实体肿瘤疗效评估标准1.1版（Response Evaluation Criteria In Solid Tumors version 1.1, RECIST v1.1），主要依据影像学上肿瘤大小的变化作为判定标准，但免疫治疗后独特的反应模式可能会低估免疫治疗对患者的获益^[76]^。

2014年欧洲肿瘤内科学会首次提出实体肿瘤免疫相关疗效评价标准（immune-related response evaluation criteria in solid tumors, irRECIST），该标准延用了RECIST v1.1标准的单径测量法，同时可测量新病灶计入总肿瘤负荷，其准确性和有效性仍有待验证。2017年初，RECIST工作组正式提出实体肿瘤免疫疗效评价标准（Modified RECIST 1.1 for immune-based therapeutics, iRECIST）^[77]^，iRECIST引入了亟待证实的疾病进展（immune unconfirmed progressive disease, iUPD）和已证实的疾病进展（immune confirmed progressive disease, iCPD）的概念，将之前RECIST v1.1评定的PD暂视为iUPD，依据患者的肿瘤类型、疾病分期和临床情况综合判断是否继续治疗，在4周-8周再次评估以确认iCPD。在此评价模式下，iUPD之后可出现免疫疾病稳定（immune stable disease, iSD）、免疫部分缓解（immune partial response, iPR）或免疫完全缓解（immune complete response, iCR），只要iCPD未证实，就需循环持续评价并记录未证实的原因。iRECIST标准提出了循环反复评价的模式，一定程度上可反映免疫治疗非典型缓解类型（如假性进展和延迟反应）。

## 7 免疫治疗超进展（hyperprogressive disease, HPD）

HPD是相对于通常的疾病进展而言，定义为肿瘤反常的加速生长，包括：①在ICIs治疗后第一次评价时出现进展，或至治疗失败时间（time to treatment failure, TTF） < 2个月；②肿瘤体积增加 > 50%；③肿瘤增长速度（tumor growth rate, TGR）增加 > 2倍。HPD的发生率约为10%，可发生于ICIs治疗过程中任何阶段，关于HPD的机制尚不明确。目前尚未发现HPD与肿瘤负荷、肿瘤类型、治疗线数、PD-L1表达水平之间存在相关性，有研究^[78, 79]^表明老年患者HPD发生率更高。发生HPD的患者总体预后较差，OS仅为3个月-4个月；一旦出现HPD的症状，需尽早由免疫治疗转为其他抗肿瘤方案。

## 8 免疫治疗的AE

以ICIs为代表的免疫治疗改变了肿瘤治疗的格局，但免疫治疗在带来生存获益的同时也会伴有AE的发生，尤其是免疫相关性AE（immune-related AEs, irAEs）^[80, 81]^。一项针对不同肿瘤组织类型及采用不同ICIs治疗后irAEs表现与发生率差异的系统评价显示，最常见的irAEs多累及内分泌器官（如甲减、甲亢、垂体和肾上腺功能障碍）、胃肠道（如腹泻、结肠炎、恶心）、肺脏（如肺炎）、皮肤（如皮疹、瘙痒、白癜风）和骨骼肌肉系统（如关节痛、肌痛）。对ICIs治疗黑色素瘤与NSCLC irAEs的比较分析显示，结肠炎、腹泻、瘙痒、皮疹等胃肠道与皮肤irAEs的发生率在黑色素瘤中较高，而肺炎等肺部irAEs的发生率在NSCLC中较高，这可能与NSCLC患者合并慢性阻塞性气道疾病或接受过肺部放疗等既往治疗有关^[82]^。虽然irAEs的总体发生率较低，但有些可致严重后果^[83]^，需高度重视和积极防治。

### 8.1 irAEs的发生机制

irAEs的发生可能与ICIs改变了机体的免疫状态有关。CTLA-4通路在T细胞反应的启动阶段起抑制作用，激活淋巴结内T细胞，同时影响Treg等细胞的功能，故CTLA-4抗体引起的irAEs较广泛、发生率较高，特异性较小，毒性较强。PD-（L）1抑制剂在T细胞效应阶段发挥作用，主要激活外周组织中的T细胞，因此引起的irAEs较CTLA-4抑制剂发生率低，且特异性较强。肿瘤免疫治疗可增强机体自身免疫系统的活性，所以ICIs除作用于肿瘤细胞之外，也会潜在地对健康组织产生毒性作用，从而引起其他系统的irAEs^[84]^。同时使用CTLA-4和PD-（L）1抑制剂，毒性会显著增加。

### 8.2 irAEs的处理原则

2018年美国临床肿瘤学会（American Society of Clinical Oncology, ASCO）联合美国国家综合癌症网络（National Comprehensive Cancer Network, NCCN）共同发布了《免疫治疗相关不良反应管理指南》以指导临床实践^[85]^，2019年CSCO发布了《CSCO免疫检查点抑制剂相关的毒性管理指南》^[86]^，2021年NCCN再次更新了免疫相关毒性管理指南（2021. V1）^[87]^。NCCN和CSCO irAE毒性管理指南存在部分不一致，本共识主要参考CSCO毒性指南。

irAEs的基本处理原则包括：预防、检查、评估、治疗和监测。

#### 8.2.1 预防

在治疗前、中及后对患者及其家属做好治疗相关AE的教育。临床医师必须熟悉irAEs的特点和危险因素，早期识别并及时处理以减少irAEs的持续时间和严重程度^[88]^。

#### 8.2.2 检查

在治疗开始前进行基线检查对判断irAEs的发生尤为重要。当患者用药后出现新的症状或原有症状加重，应完善体格检查、实验室检查和影像学检查等，必要时增加其他相关检查。

#### 8.2.3 评估

当患者用药后出现新症状或原有症状加重，可能为疾病进展、偶然事件或出现irAEs。需留意患者在使用ICIs前合并的基础疾病，关注患者已有症状，与基线值对比，判断是否为irAEs并评估其严重程度。

#### 8.2.4 治疗

irAEs的总体处理原则和irAEs级别有关，1级-4级irAE处理原则总体分为以下几种情况：①1级irAE毒性反应：一般均可在密切监测下继续治疗，但是神经系统及一些血液系统的毒性反应除外；②2级irAE毒性反应：出现大部分2级毒性反应时，应当停止治疗，直到症状和/或实验室指标恢复到1级毒性反应或更低水平，可适当给予糖皮质激素（初始剂量为泼尼松0.5 mg/kg/d-1 mg/kg/d或等剂量的其他激素）；③3级irAE毒性反应：应当停止治疗，并且立即使用高剂量糖皮质激素（泼尼松1 mg/kg/d-2 mg/kg/d，或甲基泼尼松龙1 mg/kg/d-2 mg/kg/d），糖皮质激素减量应持续4周-6周以上。对于某些毒性反应如果使用高剂量糖皮质激素48 h-72 h后症状未改善，可选择英夫利昔单抗或其他免疫抑制剂。当症状和/或实验室指标恢复到一级毒性反应或更低水平，可以恢复治疗，但应慎重，尤其是对于治疗早期就出现不良事件的患者；④4级irAE毒性反应：一般意味着永久停止治疗，已用激素替代疗法控制的内分泌不良事件除外。

irAEs发生的时间和累及器官有关，一般在给药后几周至几月内发生，但需注意的是irAE可发生于接受ICIs治疗的任何时间，甚至是延迟到ICIs治疗结束后。

#### 8.2.5 监测

推荐治疗结束后，定期监测，并与基线值进行对比，若怀疑发生irAEs，及时按处理原则进行处理。同时，注意激素相关AE的管理，如预防机会性感染、补钙以及护胃等处理。

#### 8.2.6 irAEs恢复后再次使用免疫治疗的原则

影响患者再次使用ICIs的关键因素取决于末次irAEs的程度、患者一般状况及是否存在其他治疗模式。若二级irAEs的症状和/或实验室指标降至一级或以下，可考虑再次使用ICIs，但需谨慎使用，特别对于出现早发irAEs的患者，不建议减少剂量。严重irAEs缓解后，患者再次接受ICIs治疗，irAEs可能再次出现，此时，应永久停用此类ICIs。

### 8.3 irAEs的分级和主要处理原则^[86-90]^

见表 7。

**表 7:** irAEs的分级和主要处理原则 irAEs classifications and main principles of management

irAEs	分级标准	治疗原则
腹泻/肠炎	G1：腹泻 < 每天4次	①继续ICIs治疗； ②对症（如止泻等）； ③如果症状持续或进展，需要进行粪便检查以除外感染性疾病
	G2：腹泻，每天4次-6次；出现腹痛、便血等	①暂停ICI治疗； ②口服泼尼松1 mg/kg/d。如48 h-72 h激素治疗无改善或加重：增加剂量至2 mg/kg/d；考虑加用英夫利西单抗
	G3：腹泻≥每天7次；剧烈腹痛，腹膜征等	①G3暂停ICIs治疗；G4永久停用ICIs治疗； ②静脉甲基泼尼松龙2 mg/kg/d，如48 h激素治疗无改善或加重，在继续应用激素的同时考虑加用英夫利西单抗。如果英夫利西单抗耐药，考虑维多珠单抗。
	G4：胃肠穿孔等症状危及生命，需要紧急干预治疗	
肝炎	G1：AST/ALT < 3倍ULN	①继续ICIs治疗； ②查HAV、HBV、HCV、CMV等指标，除外肝脏基础疾病； ③限酒
	G2：AST/ALT：3倍-5倍ULN	①暂停ICIs治疗； ②0.5 mg/kg/d-1.0 mg/kg/d泼尼松龙口服或等效剂量静脉
	G3：AST/ALT：5倍-20倍ULN	①暂停ICIs治疗； ②给予泼尼松1 mg/kg/d-2 mg/kg/d，3 d后无好转考虑加用麦考酚酯，不推荐使用英夫利西单抗
	G4：AST/ALT > 20倍ULN	①永久停用ICIs； ②给予泼尼松/甲基泼尼松龙1 mg/kg/d-2 mg/kg/d，3 d后无好转考虑加用麦考酚酯，不推荐使用英夫利西单抗
皮疹	G1：皮疹 < 10%体表面积	①对症治疗(如抗组胺药、外用类固醇激素)； ②继续ICIs治疗
	G2：皮疹占10%-30%体表面积	①继续ICIs治疗； ②口服抗组胺药止痒； ③使用中等-高强度的糖皮质激素（局部外用）。如对外用药无反应，考虑泼尼松0.5 mg/kg/d
	≥G3：皮疹 > 30%体表面积	①暂停ICIs治疗； ②外用强效类固醇激素，0.5 mg/kg/d-1 mg/kg/d泼尼松龙（若无改善则增加至2 mg/kg/d）； ③皮肤科会诊，考虑皮肤活检
反应性皮肤毛细血管增生症	G1：单个最大径≤10 mm，伴或不伴破溃出血	①继续ICIs治疗； ②易摩擦部位可用纱布保护，避免出血； ③破溃出血者可采用局部压迫止血治疗
	G2：单个最大径 > 10 mm，伴或不伴有破溃出血	①继续ICIs治疗； ②易摩擦部位可用纱布保护，避免出血； ③破溃出血者可采用局部压迫止血，或采取局部治疗措施，如激光或手术切除等，避免破溃处感染
	G3：呈泛发性，可以并发皮肤感染，可能需要住院治疗	①暂停ICIs治疗，直至毒性降至1级； ②易摩擦部位可用纱布保护，避免出血； ③破溃出血者可局部压迫止血，或采取局部治疗措施如激光或手术切除等，并发感染者给予抗感染治疗
	G4：多发和泛发，威胁生命	永久停药
肺炎	G1：无症状，病变局限于单个肺叶或 < 25%的肺实质	①基线检查：胸部CT、血氧饱和度、肝肾功能、肺功能等； ②考虑在3周-4周后复查胸部CT及肺功能，如影像学好转，密切随访并恢复治疗；如影像学进展，升级治疗方案，暂停免疫治疗；如影像学无改变，考虑继续治疗并密切随访直至出现新的症状
	G2：出现新的/或症状恶化，包括呼吸短促、咳嗽、胸痛、发热、需氧量增加。涉及多个肺叶且达到25%-50%的肺实质	①暂停ICIs治疗，直至降至≤G1； ②静滴甲基泼尼松龙，1 mg/kg/d-2 mg/kg/d，治疗48 h-72 h后，若症状改善，激素在4周-6周内按照每周5 mg-10 mg逐步减量；若症状无改善，按G3-G4反应治疗；如不能完全排除感染，需考虑加用经验性抗感染治疗； ③3周-4周后复查胸部CT； ④临床症状和影像学缓解至≤G1，免疫药物可在评估后使用
	G3：严重症状，涉及所有肺叶或 > 50%肺实质，个人自理能力受限，需吸氧	①永久停用ICIs治疗，住院治疗； ②如果尚未完全排除感染，需经验性抗感染治疗；必要时请呼吸科或感染科会诊； ③静脉滴注甲基泼尼松龙，2 mg/kg/d，酌情行肺通气治疗；激素治疗48 h后，若临床症状改善，继续治疗至症状改善至≤G1，然后在4周-6周内逐步减量；若无明显改善，可考虑接受英夫利昔单抗（5 mg/kg）静脉滴注，或吗啡麦考酚，1 g/ 次，2次/d，或静脉注射免疫球蛋白
	G4：危及生命的呼吸衰竭	
甲减	G1：无症状：只需临床或诊断性观察，无需治疗	继续ICIs治疗
	G2：有症状：需行甲状腺替代治疗	①继续ICIs治疗； ②TSH升高（> 10 *μ*IU/mL），补充甲状腺素
	G3：严重症状：个人自理能力受限，需住院治疗	
	G4：危及生命：需紧急干预	
甲亢	G1：同甲减G1	①继续ICIs治疗，如果有症状，普萘洛尔、美替洛尔或者阿替洛尔口服缓解症状； ②4周-6周后复查甲状腺功能：如果已经缓解，不需要进一步治疗；如果TSH仍然低于正常值，游离T4/总T3升高，建议行4 h或24 h摄碘率以明确是否有甲状腺机能亢进或毒性弥漫性甲状腺肿（Graves病）等
	G2：有症状：需要行甲状腺激素抑制治疗	
	G3：同甲减G3	
	G4：同甲减G4	
肾炎	G1：肌酐水平增长 > 26.5 *μ*mol/L；肌酐1.5倍-2倍ULN	①继续ICIs治疗； ②每3-7天随访肌酐和尿蛋白
	G2：肌酐2倍-3倍ULN	①暂停ICIs； ②每3-7天随访肌酐和尿蛋白； ③肾内科会诊； ④如排除其他原因，开始泼尼松0.5 mg/kg/d-1 mg/kg/d；如果降至G1，推荐应用ICIs；持续G2超过1周，使用泼尼松/甲基泼尼松龙1 mg/kg/d-2 mg/kg/d
	G3：肌酐 > 3倍ULN，或 > 353.6 *μ*mol/L G4：危及生命，需透析	①永久停用ICIs； ②需住院治疗、延长住院时间或紧急干预； ③每24 h监测肌酐和尿蛋白； ④肾内科会诊； ⑤泼尼松/甲基泼尼松龙，1 mg/kg/d-2 mg/kg/d；如应用激素1周后仍 > G2，考虑加用：硫唑嘌呤/环磷酰胺/环孢霉素/英夫利西单抗/霉酚酸
irAEs：免疫相关不良反应；ICIs：免疫检查点抑制剂；ALT：谷丙转氨酶；AST：谷草转氨酶；TSH：促甲状腺激素；ULN：正常值上限；CT：计算机断层扫描；HAV：甲型肝炎病毒；HBV：乙型肝炎病毒；HCV：丙型肝炎病毒；CMV：巨细胞病毒		

## 9 免疫治疗在特殊人群中的应用

### 9.1 高龄和体弱患者

对高龄晚期NSCLC患者而言，ICIs治疗的疗效和总人群类似，且安全可耐受。≥70岁患者接受纳武利尤单抗治疗后，OS获益与总人群无差异（mOS：10.4个月*vs*9.1个月；2年OS：25% *vs*26%），3级-5级严重AE（6% *vs*6%）和治疗相关AE（treatment-related adverse event, TRAE）的发生率（38% *vs*37%）与总人群无统计学差异^[91]^。另一项KEYNOTE-010/024/042研究^[92]^的汇总分析结果显示，帕博利珠单抗单药相对于化疗在PD-L1表达阳性老年（≥75岁）患者中具有与总人群类似的临床获益，且TRAE较化疗少（任何级别：68.5% *vs*94.3%；3级及以上：24.2% *vs*61.0%）。

对于不同体力活动状态评分（performance status, PS）患者的治疗，PS 0分-1分的患者可以耐受放化疗；2分可考虑接受靶向治疗或者ICIs治疗；3分-4分则需要慎重接受治疗。尽管PS 2分患者较PS 0分-1分患者生存预后更差^[91, 93]^，但仍能从ICIs治疗中获益，且安全可耐受。一项多中心单臂Ⅱ期PePS2研究^[94]^显示，PS 2分的NSCLC患者接受一线帕博利珠单抗治疗后持续临床获益率（durable clinical benefit, DCB）达38%，接受二线及以上帕博利珠单抗治疗的DCB为36%。PD-L1表达越高，临床获益越明显，DCB在PD-L1 < 1%、1-49%和≥50%的患者中分别为22%、47%和53%，mPFS分别为3.7个月、8.3个月和12.6个月，mOS分别为8.1个月、12.6个月和14.6个月。安全性方面，28%的PS 2分患者治疗后出现AE，18%出现延迟给药，10%暂停治疗，无5级TRAE，无超进展早期死亡病例。

综上所述，单纯高龄并不影响ICIs的疗效和安全性，PS 2分也并不是ICIs治疗的禁忌证。尽管PS 2分患者的总体生存仍不理想，但仍可从ICIs治疗中获益。

### 9.2 脑转移和肝转移患者

根据KEYNOTE-001/010/024/042研究汇总分析显示，对于PD-L1 TPS ≥50%伴脑转移的患者，帕博利珠单抗单药较化疗仍有OS获益（mOS：19.7个月*vs*9.7个月，HR=0.78）^[95]^。根据KEYNOTE-021/189/407研究中基线伴稳定脑转移的NSCLC患者结局的汇总分析显示，帕博利珠单抗联合化疗较单纯化疗OS获益明显（mOS：18.8个月*vs*7.6个月，HR=0.48）^[96]^。评估帕博利珠单抗一线治疗伴脑转移的黑色素瘤或NSCLC患者的Ⅱ期研究的中期分析显示，伴脑转移的NSCLC患者缓解率33%，mOS达7.7个月^[97, 98]^。

KEYNOTE-189研究中对比了帕博利珠单抗联合化疗或化疗对伴肝转移的非鳞状NSCLC的疗效，结果显示帕博利珠单抗联合化疗方案治疗后mPFS（6.1个月*vs*3.4个月，HR=0.52）和mOS均能获益（12.6个月*vs*6.6个月，HR=0.62）。IMpower150研究中对肝转移亚组的分析显示，ABCP相较于BCP方案的mPFS（8.2个月*vs*5.4个月，HR=0.41）和mOS（13.3个月*vs*9.4个月，HR=0.52）有显著改善^[99]^。

### 9.3 自身免疫性疾病（autoimmune disease, AID）

多数NSCLC合并AID的患者被排除在免疫治疗的临床研究以外，因此，ICIs治疗这类患者的安全性尚未完全清楚。AID可根据疾病控制的情况分为三类：①目前处于活动期；②目前处于治疗期且病情得到控制；③目前未治疗但病情已得到控制。一项汇总了56例NSCLC合并AID的患者接受PD-1抑制剂治疗的研究^[100]^显示，有近1/4的患者至少出现了一次AID的复发，但复发一般都是低级别的，且控制较好，不需要增加或升级免疫抑制药物；ICIs应用于第一类患者AID发作的风险较高，虽然发作仍可控制，但对这类患者使用ICIs治疗时应谨慎。在另一项回顾性研究^[101]^中，接受ICIs治疗的112例NSCLC合并AID患者中71%出现AID的爆发和/或irAE，但多数病情可控，这部分患者的PFS较无AID爆发或发生irAEs的患者更短。一项系统性回顾研究显示，75%接受ICIs治疗的合并AID的NSCLC患者会发生AID加重和/或irAEs，但多数应用糖皮质激素后可控且无需中断免疫治疗^[102]^。总之，对于合并AID的NSCLC患者应用ICIs需要多学科讨论、权衡利弊，如果病情已经控制得当，接受ICIs的治疗可能是安全的，但需密切监测，对于病情尚未控制的人群，使用ICIs大概率会加重病情。

### 9.4 长期使用激素者

已有研究^[103]^表明基线使用大剂量皮质类固醇（≥10 mg泼尼松）与免疫治疗的不良疗效相关。有研究分析了640例接受单药PD-（L）1抑制剂治疗的晚期NSCLC患者，其中90例患者（14%）在接受PD-（L）1治疗前就已开始使用皮质类固醇（≥10 mg泼尼松），分析显示这14%接受皮质类固醇（≥10 mg泼尼松）治疗的患者的OS和PFS明显变差。目前，尚不清楚这些患者疗效变差是否与使用皮质类固醇的免疫抑制作用直接相关，但建议在开始免疫治疗时谨慎使用皮质类固醇，除非需要进行激素治疗（如脑转移）。

### 9.5 肝炎病毒感染患者

合并肝炎病毒感染的肺癌患者接受ICIs治疗的数据较少。一项回顾性研究^[104]^结果显示，慢性乙肝（*n*=16）或慢性丙肝（*n*=5）的患者ICIs治疗后整体ORR为35%，mPFS为4.5个月。患者肝功能指标有轻度升高，未见3级及以上肝脏irAEs，也无患者需终止免疫治疗或应用糖皮质激素治疗AE，病毒载量无明显异常，也未见病毒再次激活。因此，慢性肝炎并非使用免疫治疗的绝对禁忌证，但需前瞻性研究作进一步的探索。

### 9.6 人类免疫缺陷病毒（human immunodeficiency virus, HIV）感染患者

HIV病毒感染会严重损害机体免疫系统，因此各类肿瘤的发生率明显提高。另一方面，PD-（L）1抑制剂本身并不能直接杀死肿瘤细胞，而是通过激活人体的免疫系统发挥作用，因此PD-（L）1抑制剂需要一个基本完整的免疫系统，才能发挥抗肿瘤的作用。有研究^[105]^分析了30例合并HIV病毒感染的肿瘤患者接受PD-（L）1抑制剂治疗，结果显示，在安全性方面，22例患者出现了1级-2级较轻微AE，6例患者出现了3级AE，而AE的表现形式与普通人群基本相同，包括乏力、甲减、恶心、皮疹、肺炎等；在疗效方面，1例肺癌患者肿瘤完全消失，2例淋巴瘤患者肿瘤明显缓解，2例卡波西肉瘤患者疾病稳定，总体的抗肿瘤疗效和普通人群无明显差异。治疗期间HIV病毒数量未出现明显的反弹，病情未处于活动，但CD4^+^ T细胞数量也未出现明显的恢复。另一项系统评价^[106]^纳入了73例接受免疫治疗的HIV感染患者，发现3级以上AE发生率为8.6%，NSCLC患者ORR达30%，93%的患者持续保持低HIV病毒载量，CD4^+^ T细胞数量稳定。ICIs治疗HIV感染的晚期恶性肿瘤患者疗效及安全性的Ⅱ期DURVAST临床研究中，所有患者入组时均接受抗逆转录病毒治疗，结果表明，50%的患者出现药物相关AE，但均为1级-2级，ORR和DCR分别为25%和56%。CD4^+^和CD8^+^ T细胞计数和血浆HIV病毒载量维持稳定^[107]^。因此，对于病情基本控制的HIV感染患者来说，免疫治疗仍然可能是有效的方案。

## 附录：ICIs一线及二线治疗晚期NSCLC Ⅲ期临床研究释义

KEYNOTE-021G研究在晚期非鳞状NSCLC患者中评估了帕博利珠单抗联合培美曲塞/卡铂对比培美曲塞/卡铂的疗效^[108]^。结果显示，帕博利珠单抗联合组ORR和OS获益显著（ORR：58% *vs*33%；mOS：34.5个月*vs*21.1个月，HR=0.71；3年生存率：50% *vs*37%）。Ⅲ期研究KEYNOTE-189对比了帕博利珠单抗联合培美曲塞/铂类较安慰剂联合培美曲塞/铂类治疗在晚期EGFR/ALK野生型非鳞NSCLC患者的疗效和安全性。免疫联合组展现出绝对的疗效优势，mOS：22.0个月*vs*10.6个月（HR=0.60），3年OS率：31.3% *vs*17.4%，mPFS：9.0个月*vs*4.9个月（HR=0.50）。两种治疗方案的AE相当，≥3级TRAE发生率分别为52.1% *vs*42.1%，均可控。值得一提的是，不论PD-L1表达状态如何，免疫联合组患者生存均明显延长^[19, 20, 109]^。FDA及NMPA分别于2017年和2019年批准了帕博利珠单抗联合含铂双药用于晚期无驱动基因突变的非鳞NSCLC患者的一线治疗。IMpower130研究在晚期非鳞状NSCLC患者中评估了阿替利珠单抗联合白蛋白紫杉醇/卡铂对比白蛋白紫杉醇/卡铂的疗效，结果显示免疫联合化疗组显著延长mPFS（7.0个月*vs*5.5个月，HR=0.64）和mOS（18.6个月*vs*13.9个月，HR=0.79）^[21]^。2019年FDA批准阿替利珠单抗联合白蛋白紫杉醇/卡铂用于EGFR/ALK阴性转移性非鳞状NSCLC的一线治疗。CameL研究评估了卡瑞利珠单抗联合培美曲塞/卡铂对比单纯化疗一线治疗晚期非鳞状NSCLC的疗效和安全性，结果显示联合卡瑞利珠单抗显著延长mPFS（11.3个月*vs*8.3个月，HR=0.60）^[22, 23]^。2020年NMPA批准卡瑞利珠单抗联合培美曲塞/卡铂用于EGFR/ALK阴性的、不可手术切除的局部晚期或转移性非鳞状NSCLC的一线治疗。ORIENT-11研究对比了信迪利单抗联合培美曲塞/铂类对比单纯化疗一线治疗EGFR/ALK阴性晚期非鳞状NSCLC的疗效和安全性，结果显示联合信迪利单抗显著延长mPFS（8.9个月*vs*5.0个月，HR=0.48）和mOS（未达vs未达，HR=0.61）^[24]^。2021年NMPA批准信迪利单抗联合培美曲塞/铂类一线治疗非鳞状NSCLC。RATIONALE 304研究结果显示，替雷利珠单抗联合培美曲塞/铂类较单纯化疗一线治疗晚期非鳞状NSCLC，显著延长mPFS（9.7个月*vs*7.6个月，HR=0.645）^[25]^。GEMSTONE-302研究结果显示，舒格利单抗联合培美曲塞/卡铂对比单纯化疗一线治疗Ⅳ期非鳞状NSCLC，显著延长mPFS（8.57个月*vs*5.16个月，HR=0.66）^[26]^。

KEYNOTE-407研究评估了帕博利珠单抗联合紫杉醇或白蛋白紫杉醇/卡铂对比化疗一线治疗晚期鳞癌NSCLC患者的疗效和安全性。与单纯化疗相比，帕博利珠单抗联合化疗组显著改善OS（mOS：17.1个月*vs*11.6个月，HR=0.71；2年生存率：37.5% *vs*30.6%），在PD-L1 < 1%、1%-49% 和≥50%的人群中，死亡风险分别降低21%、41%和21%，不同PD-L1表达人群均有获益^[27-29]^。2018年美国FDA批准了帕博利珠单抗联合紫杉醇或白蛋白紫杉醇/卡铂一线治疗晚期鳞状NSCLC。KEYNOTE-407中国扩展研究同样证实了帕博利珠单抗联合化疗相对于单纯化疗改善了mOS（17.3个月*vs*12.6个月，HR=0.44）和mPFS（8.3个月*vs*4.2个月，HR=0.32）^[30]^。NMPA已于2019年批准该方案一线治疗转移性鳞状NSCLC。RATIONALE 307研究^[31]^显示，替雷利珠单抗联合紫杉醇或白蛋白紫杉醇/卡铂一线治疗晚期鳞状NSCLC较单纯化疗显著改善mPFS（7.6/7.6个月*vs*5.5个月），HR分别为0.52和0.48。2021年NMPA批准替雷利珠单抗联合紫杉醇或白蛋白紫杉醇/卡铂一线治疗晚期鳞状NSCLC。ORIENT-12研究^[32]^显示，信迪利单抗联合吉西他滨/铂类较化疗一线治疗鳞状NSCLC能显著延长mPFS（5.5个月*vs*4.9个月，HR=0.54）。GEMSTONE-302研究^[26]^显示，舒格利单抗联合紫杉醇/卡铂对比单纯化疗一线治疗Ⅳ期鳞状NSCLC，显著延长mPFS（7.16个月*vs*4.70个月，HR=0.33）。

KEYNOTE-024研究显示帕博利珠单抗在PD-L1≥50%的驱动基因阴性晚期NSCLC人群中，帕博利珠单抗治疗较标准含铂化疗的PFS（HR=0.50）与OS（HR=0.62）都得到了显著改善，帕博利珠单抗组5年生存率达31.9%，明显高于化疗组（16.3%），且任何级别的TRAE（76.6% *vs*90.0%）和≥3级的TRAE发生率少于化疗组（31.2% *vs*53.3%）^[33, 34]^。2016年FDA批准帕博利珠单抗用于PD-L1≥50%的驱动基因阴性晚期NSCLC的一线治疗。KEYNOTE-042研究^[67]^进一步探索了帕博利珠单抗单药一线治疗PD-L1≥1%的NSCLC患者的效果，结果显示帕博利珠单抗组的mOS均优于单独化疗组，其中PD-L1≥50%的人群的疗效最为显著。随访数据^[35]^显示，PD-L1≥1%的患者经帕博利珠单抗单药一线治疗的3年OS率可达25%。KEYNOTE-042中国人群数据同样证实了一线帕博利珠单抗单药较化疗在各PD-L1表达（≥50%; ≥20%; ≥1%）人群中均有mOS获益（≥50%：24.5个月*vs*13.8个月，HR=0.63；≥1%：20.2个月*vs*13.5个月，HR=0.67），反应持续时间（duration of response, DOR）超15个月，且安全性可控^[36]^。该研究将帕博利珠单抗治疗的优势人群由PD-L1≥50%扩展至PD-L1≥1%的驱动基因阴性晚期NSCLC人群，2019年FDA和NMPA批准了帕博利珠单抗单药一线治疗适应证。IMpower110研究探索了阿替利珠单抗相比铂类联合培美曲塞或吉西他滨用于经PD-L1筛选的驱动基因阴性Ⅳ期NSCLC初治患者的疗效和安全性，结果显示阿替利珠单抗较化疗显著改善TC3/IC3患者的mOS（20.2个月*vs*14.7个月，HR=0.76）^[37, 38]^。2020年FDA批准阿替利珠单抗用于一线治疗NSCLC，限用于TC3/IC3人群。CheckMate 026研究探索了纳武利尤单抗对比含铂化疗一线治疗PD-L1≥5%的晚期NSCLC，结果显示纳武利尤单抗相比标准化疗未能延长PFS和OS^[39]^。并非所有的免疫单药作为驱动基因阴性的NSCLC一线治疗均能使患者获益，临床实践中还需要根据临床研究的数据及国内获批适应证的情况进行合理选择。

IMpower150研究是探索抗血管生成治疗结合化疗的基础上联合免疫治疗的疗效，对比阿替利珠单抗+贝伐珠单抗+卡铂+紫杉醇（ABCP）、阿替利珠单抗+卡铂+紫杉醇（ACP）与标准治疗贝伐珠单抗+卡铂+紫杉醇（BCP）用于未经化疗的转移性非鳞状NSCLC患者的疗效及安全性。结果显示，ABCP组（阿替利珠单抗+贝伐珠单抗+卡铂+紫杉醇）对比BCP组（贝伐珠单抗+卡铂+紫杉醇）OS获益明显（19.5个月*vs*14.7个月，HR=0.80），而ACP方案（阿替利珠单抗+卡铂+紫杉醇）未显著优于BCP方案^[40, 41]^。2018年FDA批准ABCP方案用于转移性非鳞状NSCLC的一线治疗。ABCP方案同时也增加了3级-4级AE发生率，临床应用时需要充分评估获益及潜在风险。

CheckMate 227研究Ia部分结果显示，纳武利尤单抗联合伊匹单抗的一线双免治疗较化疗能显著改善PD-L1≥1%的晚期NSCLC患者OS（mOS：17.1个月*vs*14.9个月，HR=0.79），3年生存率达33%^[42]^。2020年FDA批准了纳武利尤单抗联合伊匹单抗的双免疫组合方案治疗驱动基因阴性的PD-L1≥1%的晚期NSCLC。CheckMate 9LA研究^[44]^结果显示，无论PD-L1表达水平，纳武利尤单抗和伊匹单抗的双免疫联合加含铂化疗对比化疗一线治疗晚期NSCLC能显著延长OS（15.6个月*vs*10.9个月，HR=0.66）和PFS（6.7个月*vs*5.0个月，HR=0.68）。2020年FDA批准纳武利尤单抗和伊匹单抗联合2周期含铂化疗一线治疗EGFR/ALK阴性晚期NSCLC。考虑到KEYNOTE 598研究在PD-L1 TPS≥50%人群中显示帕博利珠单抗联合伊匹单抗未优于帕博利珠单抗单药治疗，在该人群中选择PD-1（L1）联合CTLA-4的双免疫方案需要慎重^[43]^。

KEYNOTE-001研究探索帕博利珠单抗治疗晚期NSCLC的疗效与安全性。数据显示PD-L1表达与帕博利珠单抗疗效相关，帕博利珠单抗治疗安全性可控^[110]^。在此研究基础上，KEYNOTE-010研究纳入PD-L1≥1%且既往接受过至少一种化疗方案的局部晚期或转移性NSCLC患者，对比帕博利珠单抗与多西他赛的疗效。结果显示，无论是帕博利珠单抗标准剂量2 mg/kg组还是高剂量10 mg/kg组的OS，均明显优于多西他赛组（10.4个月*vs*12.7个月*vs*8.5个月）。随访结果显示，PD-L1≥50%的患者接受帕博利珠单抗治疗较化疗OS明显延长（mOS：16.9个月*vs*8.2个月，HR=0.55；5年OS率：25.0% *vs*8.2%）。PD-L1≥1%的患者中，同样也观察到了帕博利珠单抗治疗的OS获益，5年OS率可达15.6%^[45, 46]^。基于上述研究，FDA批准了帕博利珠单抗二线治疗既往接受过至少一种化疗的PD-L1≥1%的局部晚期或转移性NSCLC患者。KEYNOTE-033研究评估了帕博利珠单抗对比多西他赛二线治疗中国晚期NSCLC患者的疗效，在PD-L1≥50%的人群中，OS未达统计学显著性，在PD-L1≥1%的人群中，帕博利珠单抗依然显示了OS的获益趋势^[111]^。CheckMate 017、CheckMate 057和CheckMate 078三项Ⅲ期研究显示了纳武利尤单抗单药用于二线治疗接受过含铂化疗方案的驱动基因阴性的NSCLC患者的疗效。在晚期鳞癌中，纳武利尤单抗单药较多西他赛显著改善mOS（9.2个月*vs*6.0个月，HR=0.62）^[51, 52]^。在晚期非鳞癌中，纳武利尤单抗单药较多西他赛也能改善mOS（12.2个月*vs*9.5个月，HR=0.70）^[52, 53]^。在中国晚期鳞癌与非鳞癌患者中，纳武利尤单抗仍然优于多西他赛（mOS：11.9个月*vs*9.5个月，HR=0.75）^[49, 50]^，且三项研究中≥3级的AE的发生率纳武利尤单抗明显低于化疗组^[49, 112]^。FDA及NMPA分别于2015和2018年批准纳武利尤单抗用于治疗驱动基因阴性的晚期NSCLC的二线治疗。POPLAR研究（Ⅱ期）^[113]^和OAK研究（Ⅲ期）^[47, 48]^分别评估了PD-L1抗体阿替利珠单抗二线治疗复发性局部晚期或转移性NSCLC的患者的疗效和安全性。研究显示，与多西他赛治疗组相比，阿替利珠单抗可显著提高患者的mOS（POPLAR：12.6个月*vs*9.7个月，HR=0.76；OAK：13.3个月*vs*9.8个月，HR=0.78）^[48]^。2016年，FDA批准阿替利珠单抗单药二线治疗晚期NSCLC，无论PD-L1的表达水平。JAVELIN Lung 200研究中单药Avelumab作为二线疗法治疗晚期PD-L1阳性NSCLC较多西他赛并未获得改善OS的阳性结果^[114]^。
